# Influence of the Hinge Region and Its Adjacent Domains on Binding and Signaling Patterns of the Thyrotropin and Follitropin Receptor

**DOI:** 10.1371/journal.pone.0111570

**Published:** 2014-10-23

**Authors:** Jörg Schaarschmidt, Sandra Huth, René Meier, Ralf Paschke, Holger Jaeschke

**Affiliations:** 1 Department of Internal Medicine, Neurology and Dermatology, Division of Endocrinology and Nephrology, University of Leipzig, Leipzig, Germany; 2 Institute of Biochemistry, University of Leipzig, Leipzig, Germany; Duke University Medical Center, United States of America

## Abstract

Glycoprotein hormone receptors (GPHR) have a large extracellular domain (ECD) divided into the leucine rich repeat (LRR) domain for binding of the glycoprotein hormones and the hinge region (HinR), which connects the LRR domain with the transmembrane domain (TMD). Understanding of the activation mechanism of GPHRs is hindered by the unknown interaction of the ECD with the TMD and the structural changes upon ligand binding responsible for receptor activation. Recently, our group showed that the HinR of the thyrotropin receptor (TSHR) can be replaced by those of the follitropin (FSHR) and lutropin receptor (LHCGR) without effects on surface expression and hTSH signaling. However, differences in binding characteristics for bovine TSH at the various HinRs were obvious. To gain further insights into the interplay between LRR domain, HinR and TMD we generated chimeras between the TSHR and FSHR. Our results obtained by the determination of cell surface expression, ligand binding and G protein activation confirm the similar characteristics of GPHR HinRs but they also demonstrate an involvement of the HinR in ligand selectivity indicated by the observed promiscuity of some chimeras. While the TSHR HinR contributes to specific binding of TSH and its variants, no such contribution is observed for FSH and its analog TR4401 at the HinR of the FSHR. Furthermore, the charge distribution at the poorly characterized LRR domain/HinR transition affected ligand binding and signaling even though this area is not in direct contact with the ligand. In addition our results also demonstrate the importance of the TMD/HinR interface. Especially the combination of the TSHR HinR with the FSHR-TMD resulted in a loss of cell surface expression of the respective chimeras. In conclusion, the HinRs of GPHRs do not only share similar characteristics but also behave as ligand specific structural and functional entities.

## Introduction

Glycoprotein hormone receptors (GPHRs) are mediators of signal transduction for important biological processes such as reproduction and thyroid physiology. A dysfunction of these receptors, *e.g*. by *in vivo* mutations, can lead to severe pathological effects [1]–[3]. Therefore, understanding the mechanism of action of GPHRs is an important tool in the development of ligands with specific binding and signaling profiles to treat various diseases mediated by GPHRs.

**Figure 1:**
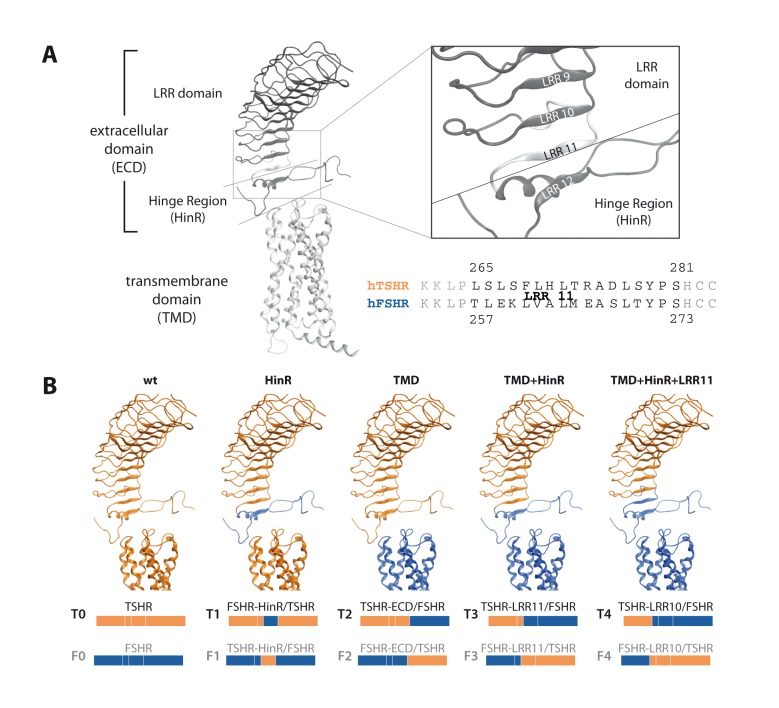
Components of glycoprotein hormone receptors (GPHRs) and composition of the generated receptor chimeras. A: GPHRs consist of a transmembrane domain common to all GPCR and an extracellular domain (ECD), which can be further subdivided into a leucine-rich repeat (LRR) domain and the hinge region (HinR). Previous crystal structures included only the first 10 LRRs. The Alignment shows the protein sequence of the 11^th^ LRR of the TSHR and the FSHR, which can, based on the chosen HinR definition, considered to be part of the LRR domain or the HinR. B: Composition and terminology of the receptor chimeras. The structural representation shows the chimeras including the LRR domain of the TSHR. Components with TSHR protein sequence are colored in orange and components with FSHR protein sequence are colored in blue.

GPHRs are a subfamily of class A (rhodopsin-like) G protein coupled receptors (GPCRs) [4]. Members of this subfamily are the thyrotropin receptor (TSHR), the lutropin/choriogonadotropin receptor (LHCGR) and the follitropin receptor (FSHR) [5]. A key feature of GPHRs is the large extracellular domain (ECD) responsible for ligand binding. (Fig. 1A, left panel). The endogenous ligands of GPHRs, the glycoprotein hormones (GPHs), consist of a common alpha subunit and a receptor specific beta-subunit and binding of these ligands lead to the activation of intracellular G proteins via conformational changes in the TMD [6]. The ECD of GPHRs is subdivided into two parts: (I) a leucine rich repeat (LRR) domain and (II) the hinge region (HinR), which connects the LRR domain with the TMD. While the LRR domain of GPHRs is believed to be exclusively responsible for ligand binding, the HinR is considered to be involved in binding and signaling as well [7]. The extracellular portion of the receptor furthermore includes three extracellular loops (ECLs) connecting the transmembrane helices of the TMD.

Recently, Jiang and co-workers published a crystal structure of the entire FSHR ectodomain including the majority of the HinR. This structure showed that instead of being a separate structural unit, the HinR forms a continuous structure with the LRR domain by contributing two further LRRs connected by a long protruding loop structure (Fig. 1A, right panel) [8]. Earlier mutagenesis studies have shown that the HinR contains important sites for ligand binding, like the mandatory sulfated tyrosine, which is conserved within GPHRs [9]–[11] or negatively charged residues in the TSHR HinR involved in the binding of superagonists [12], [13]. However, the HinR is the most variable part of GPHRs in length and amino acid composition [7]. Therefore it may also be least structurally and functionally conserved within GPHRs. The TSHR HinR shows the greatest deviation in length with a cleavable 50 amino acid insertion (C peptide) not present in FSHR and LHCGR. In contrast to the FSHR and LHCGR the mature TSHR therefore consists of two disulfide-linked subunits due to excision of the C peptide [14]–[16]. Up to date a physiological or functional role of the cleavage process is still unknown [17].

The interplay between the HinR and ECLs is an integral part of signal generation and transduction [18]–[20]. For the TSHR and FSHR the HinR is considered to act as an inverse agonist, which turns into an agonist of the TMD after ligand binding. However, inverse agonism of the HinR in the basal receptor state for the LHCGR could not be confirmed while agonistic properties as shown for the TSHR and the FSHR are also present [18], [21]–[24]. Even though the new crystal structure [8] provided new insights into structural and functional features of GPHRs, the most important aspects concerning the interplay of the HinR with the TMD including the structural changes triggered upon hormone binding remain unanswered.

Chimeras have previously been employed in investigating the structure-function relationship of GPHRs and potentially help identify receptor-specific interacting regions [25]–[28]. Recently, our group showed that the HinR of the thyrotropin receptor (TSHR) can be replaced by those of the follitropin (FSHR) and lutropin receptor (LHCGR) without effects on cell surface expression and hTSH signaling [26]. However, in an earlier study, Nagayama et al. [28] showed that no binding and signaling of hCG is observed if the TSHR LRR domain is replaced by the LHCGR LRR domain and that bovine TSH is incapable of activating the Gs pathway of this chimera despite of high affinity binding. While our results confirm that the general mechanism of binding, especially to the sulfotyrosine in the HinR [8]–[10], as well as signal initiation and propagation is similar in all three GPHRs, the data of Nagayama et al. [28] suggest that receptor specific properties exist that impede the proper function of the TSHR HinR when combined with LHCGR LRR domain. To further investigate these receptor-specific differences as well as their likely location within the receptor, we generated various chimeras between the TSHR and FSHR since chimeras between these two receptors have not been explored in detail (Fig. 1B).

## Materials and Methods

### Generation of TSHR constructs

Constructs were generated using standard PCR techniques. Briefly, cDNA's for constructs were amplified by overlap-extension PCR using templates hTSHR(HA)-pcDNA3.1/Zeo^(−)^ and hFSHR(HA)- pcDNA3.1/Zeo^(−)^ (www.cDNA.org). The length of the HinR was defined according to Kleinau et al. [29].

### Cell culture and transient expression of mutant TSHRs

COS-7 cells [30] were grown in high glucose Dulbecco's modified Eagle's medium (DMEM; PAA Laboratories, Pasching, Austria) supplemented with 10% FCS, 100 U/ml penicillin and 100 µg/ml streptomycin (Gibco Life technologies, Paisley, UK) at 37°C in a humidified 5% CO_2_ incubator. Cells were transiently transfected in 12-well plates (1×10^5^ cells per well) or 24-well plates (0.5×10^5^ cells per well) with 1 µg and 0.5 µg DNA per well, respectively, using the GeneJammer Transfection Reagent (Stratagene, Amsterdam, NL).

### Determination of cell surface expression

Determination of the receptor's cell surface expression was performed as previously described by Müller et al. [31]. For permeabilized cell assay, in the first step cells were fixed with 1% paraformaldehyde for 10 min on ice following incubation with PBS containing 0.1% bovine serum albumin and 0.2% saponin for 30 min. Saponin was supplemented in all subsequent buffers. Subsequently, cells were incubated for 1 h with a 1∶400 dilution of a mouse anti-HA antibody (Sigma). Cells were washed twice and incubated for 1 h with a 1∶400 dilution of an Alexa488 conjugated rabbit anti-mouse antibody (Invitrogen). Before FACS analysis (FACscan; BD Biosciences), cells were washed twice and fixed with 1% paraformaldehyde. Receptor expression was determined by fluorescence intensity; the percentage of signal positive cells corresponded to transfection efficiency.

The cell surface expression of all characterized chimeras were normalized to the wild type receptor containing the respective transmembrane domain.

### Determination of intracellular cyclic AMP accumulation

For the determination of intracellular cAMP concentrations, cells were incubated in serum free DMEM (PAA Laboratories, Pasching, Austria) supplemented with 1 mM 3-isobutyl-1-methylxanthine (IBMX; Sigma) for one hour and accumulated cAMP concentrations were determined as previously described by Müller et al. [31]. For stimulation curves the medium was supplemented with increasing concentrations of bovine (b) TSH (0–30 mU/ml), recombinant human (rh) TSH (0–100 mU/ml), rhFSH (0–1000 ng/ml) or human (h) FSH analog TR4401 (0–1000 ng/ml). Highly purified bTSH (30 U/mg) was purchased from Dr. A. Parlow and the NIDDK National Hormone and Pituitary Program and reconstituted to 30 U/ml. rhTSH (∼8 U/mg; Thyrogen) was purchased from Genzyme (Neu-Isenheim, Germany) and reconstituted to ∼8 U/ml. rhFSH (44 µg/ml; Gonal-F) was purchased from Merck Serono (Darmstadt, Germany). hFSH analog TR4401 was kindly provided from Dr. Mariusz Szkudlinski (Trophogen, Rockville, Maryland, USA) and reconstituted to 1.1 mg/ml.

### Competitive binding assay

48 hours after transfection COS-7 cells were incubated in modified Hank's buffer (5.36 mM KCl; 0.44 mM KH_2_PO_4_; 0.41 mM MgSO_4_; 0.33 mM Na_2_HPO_4_; 5.55 mM Glucose) supplemented with 1.3 mM CaCl_2_; 280 mM Sucrose; 0.2% BSA and 2.5% milk powder in the presence of 80.000 cpm/ml of ^125^I-bTSH (Thermo Fisher Scientific, B.R.A.H.M.S), or 10.000–20.000 cpm/ml ^125^I-rhFSH (Perkin Elmer) with increasing concentrations of unlabeled ligand (0–100 mU/ml bTSH – NIDDK National Hormone and Pituitary Program; 0–1000 ng rhFSH – Gonal-F, Merck Serono) at 4°C for four hours. Afterwards cells were washed with the same ice-cold buffer, solubilized with 1N NaOH, and radioactivity was measured in a gamma-counter. Specific binding was determined by subtracting the amount of radioligand unspecific bound to cells transfected with the empty pcDNA3.1/Zeo^(−)^ vector from each data set of the characterized receptor variants. The parameter maximal binding capacity was determined by nonlinear regression of competition binding curves using Graph Pad Prism 4.0 for Windows assuming a one-site binding model. The maximal binding capacity of wild type receptor was set at 100%, and the maximal binding of all mutants was calculated according to this.

### Statistical analysis

Statistical analysis was carried out using the nonparametric t test of GraphPad Prism 4.0 for Windows (p value <0.001– extremely significant, <0.01– very significant, <0.05– significant, >0.05– not significant).

### Molecular modeling

Homology models of the GPHR Chimeras ectodomains in complex with the hormones and their visual representations were generated using the Molecular Operating Environment (MOE, 2012.10; Chemical Computing Group Inc., Montreal, QC, Canada). For all homology models, the crystal structure of the FSHR ectodomain in complex with FSH (PDB code 4AY9) was used as template [8]. The protein sequences of the human TSHR and hTSH were acquired from the UniProt database (Accession number hTSHR: P16473, hTSH: P01222) [32] and chimera ectodomain sequences generated by combining the corresponding parts of TSHR and FSHR protein sequence. The sequences of the chimeras were aligned to the protein sequence of the crystal structure within MOE prior to the homology modeling step. For each chimera 100 homology models with 3 side chain samples at 300 K were generated employing the Amber12 force field. During homology modeling the hormone FSH from the crystal structure was retained as environment for all chimeras harboring the FSHR LRR domain whereas for chimeras harboring the TSHR LRR domain, only the common alpha subunit was retained as environment while the beta subunit of hTSH was modeled onto the coordinates of the FSH beta subunit.

## Results

To extend our knowledge concerning the functional impact of the HinR of GPHRs we initially generated a FSHR construct, where the HinR of the FSHR was replaced by its TSHR counterpart (Table 1, construct F1). This construct F1 (TSHR-HinR/FSHR) showed impaired cell surface expression of 6%, when compared with the wt TSHR (set at 100%, Table 1). FACS analysis with permeabilized cells revealed that this receptor chimera was trapped in the intracellular compartment, indicated by an increase of the fluorescent signal to 80% of the wt TSHR in permeabilized cells (data not shown).

**Table 1:** Functional characterization of the TSHR/FSHR chimeras.

Constructs	cell surface expression (% wt ± SD)	cAMP accumulation in nM (95% confidence intervals)									specific binding (% wt ± SD)	
			rhTSH		bTSH		rhFSH		TR4401			
		basal	100 mU/ml	EC50 in mU/ml	100 mU/ml	EC50 in mU/ml	1000 ng/ml	EC50 in ng/ml	1000 ng/ml	EC50 in mU/ml	125I -bTSH	125I -hFSH
**pcDNA3.1 (−) zeo**	2±1	1.0±0.2	2.1±0.7	–	1.7±0.3	–	1.1±0.1	–	1.1±0.2	–	3±1	2±1
**T0 – TSHR**	100	12.1 (9.6–14.5)	73.8 (71.1–76.6)	0.5 (0.4–0.7)	77.8 (75.2–80.3)	0.2 (0.2–0.3)	15.0 (12.9–17.1)	n.d.	38.3 (36.0–40.6)	n.d.	100	3±1
**T1 – FSHR-HinR/TSHR**	124±6**	7.8 (2.9–12.7)	57.2 (51.1–63.3)	0.4 (0.2–1.0)	60.8 (55.2–66.4)	2.0 (1.2–3.3)	15.2 (13.8–16.6)	n.d.	36.4 (34.9–37.9)	n.d.	28±2***	3±1
**T2 – TSHR-ECD/FSHR**	9±1***	1.7 (1.1–2.2)	9.2 (7.0–11.4)	n.d.	3.9 (3.3–4.5)	n.d.	3.5 (2.2–4.9)	n.d.	2.3 (1.6–3.0)	n.d.	n.d.	n.d.
**T3 – TSHR-LRR11/FSHR**	118±8	2.4 (1.6–3.2)	32.9 (30.7–35.1)	3.8 (2.8–5.2)	31.2 (27.9–34.4)	13.7°	2.2 (1.8–2.7)	n.d.	9.8 (8.2–11.3)	n.d.	7±1	n.d.
**T4 – TSHR-LRR10/FSHR**	131±4***	1.5 (1.0–2.0)	36.6 (33.7–39.5)	11.6 (8.6–15.6)	30.7 (27.8–33.6)	25.2°	2.0 (1.6–2.3)	n.d.	7.5 (5.7–9.2)	n.d.	7±1	n.d.
**F0 – FSHR**	100	1.7 (1.4–2.0)	2.9 (2.6–3.2)	n.d.	1.5 (1.2–1.8)	n.d.	35.3 (33.6–37.0)	5.0 (3.7–6.9)	50.0 (47.9–52.0)	1.7 (1.2–2.3)	5±1	100
**F1 – TSHR-HinR/FSHR**	6±1***	1.6 (0.6–2.8)	1.8 (1.5–2.6)	n.d.	1.4 (1.1–1.7)	n.d.	4.0 (3.1–4.8)	n.d.	2.5 (1.9–3.2)	n.d.	n.d.	n.d.
**F2 – FSHR-ECD/TSHR**	94±4	2.5 (2.0–3.1)	11.7 (9.7–13.8)	n.d.	3.7 (3.0–4.3)	n.d.	83.1 (76.1–90.1)	3.5 (2.5–5.9)	102.2 (97.6–106.9)	4.8 (3.5–6.6)	n.d.	160±6
**F3 – FSHR-LRR11/TSHR**	78±6 **	2.3 (1.9–2.7)	6.7 (5.9–7.4)	n.d.	19.3 (14.4–24.2)	n.d.	65.3 (61.3–69.4)	14.1 (9.9–20.0)	80.0 (75.1–85.0)	3.0 (1.9–4.6)	6±1***	72±2
**F4 – FSHR-LRR10/TSHR**	45±2***	4.3 (3.9–4.6)	18.5 (17.6–19.3)	n.d.	45.1 (41.2–48.2)	10.3 (7.8–13.6)	57.9 (53.7–62.1)	6.2 (3.8–10.0)	89.9 (82.6–97.2)	1.9 (1.1–3.4)	19±2***	96±2

COS-7 cells were transiently transfected with the respective DNA constructs. The cell surface expression of all characterized chimeras were normalized to the wild type receptor containing the respective transmembrane domain. Values for intracellular cAMP accumulation are given in nM and 95% confidence intervals (CI) using the GraphPad Prism 4.0 software. EC_50_ marked with ° are estimated values because dose-response curves did not reach a plateau. Data are summarized of at least three independent experiments, each carried out in duplicates. The pcDNA3.1/Zeo^(−)^ vector was used as a control. Statistical analysis for cell surface expression and specific binding capacity was carried out as described in Material and Methods. * 0.01–0.05 significant. ** 0.001–0.01 very significant. *** <0.001 extremely significant. LRR, leucine-rich repeat; HinR, hinge region; TMD, transmembrane domain; ECD, extracellular domain.

The TSHR-HinR/FSHR chimera (construct F1) includes two transitions between TSHR and FSHR protein sequence. One is located between the LRR domain of the FSHR and the HinR of the TSHR and the other one between the HinR of the TSHR and the transmembrane domain (TMD) of the FSHR. To test whether an incompatibility of the HinR with one (LRRD-HinR or HinR-TMD) or both of the adjacent domains (LRRD-HinR-TMD) is responsible for the low cell-surface expression level, we generated additional chimeras with only one transition between TSHR and FSHR protein sequence (constructs T2–4 and F2–4, Fig. 1B, Table 1).

In general we selected transition sites, where short stretches of the protein sequence were identical between the GPHRs. Because of ambiguities for the exact location of the transition from the LRR domain to the HinR and with the poorly characterized transition region at LRR 10 and 11, we generated chimeras with two different HinR definitions. While constructs T3 and F3 were generated according to Kleinau and Krause [29] with the transition site between S281 and H282 of the TSHR (FSHR S273 and H274), chimeras T4 and F4 contain the LRR/HinR transition based on the first published crystal structures for the TSHR by Sanders et al. [33] (TSHR: P264 and L265) and for the FSHR by Fan and Hendrickson [34] (FSHR: P256 and T257) (Figure 1A, Table 1). The third transition site (HinR/TMD) is located at residue D410 for the TSHR and at position D358 for the FSHR.

### Cell surface expression

Substitution of the entire extracellular domain (ECD) of the TSHR by the FSHR-ECD did not reveal significant changes in cell surface expression (construct F2, Table 1). The opposite chimera TSHR-ECD/FSHR (construct T2, Table 1) showed almost no expression at the cell surface. However, expression levels were similar to the wt TSHR in permeabilized cells (data not shown). Constructs F3 and F4 with different lengths of the FSHR-LRR domain fused to the TSHR showed a significant decline in cell surface expression to 78 and 45%, respectively (Table 1). Contrary, an increase in surface expression was observed for chimeras with various lengths of the TSHR-LRR domain fused to the FSHR ranging from 118 to 131% (constructs T3 and 4, Table 1).

### cAMP signaling

While the TSHR with the FSHR HinR (T1) exhibits a significant basal cAMP production similar to the wt TSHR, most of the chimeras do not show a significant increase in basal cAMP levels compared to mock-transfected cells. From the remaining constructs only the chimera F4 with the TMD and HinR of the TSHR and 10 LRRs of the FSHR displays a significant basal activity (Table 1).

#### Recombinant human TSH

**Figure 2:**
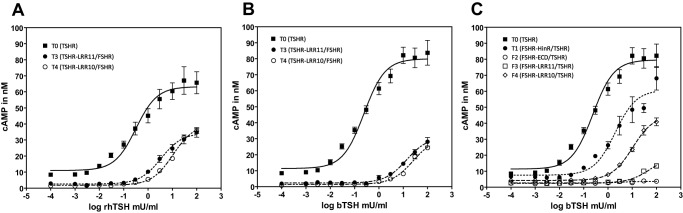
Dose-response curves of the G_s_ pathway with increasing concentrations of rhTSH (A) or bTSH (B and C). Data are presented as means ± SD of at least three independent experiments, each carried out in duplicates. EC_50_ were calculated using the GraphPad Prism 4.0 software for windows applying the nonlinear curve fit module.

Interestingly, chimeras harboring the LRR domain of the TSHR and the TMD of the FSHR showed a reduced cAMP production at 100 mU/ml rhTSH and a strong right shift of the dose response curves compared to the wt TSHR (T0), which lead to a 8-fold higher EC_50_ for construct T3 and a 23-fold higher EC_50_ for construct T4 (Table 1, Fig. 2A). A promiscuous activation by rhTSH at the highest concentrations (30–100 mU/ml) was observed for the constructs containing the FSHR LRR domain and differently sized HinRs of the TSHR (F2–4). An EC_50_ could not be determined since the dose response curves did not reach a plateau (Table 1).

#### Bovine TSH

A remarkable decrease in receptor activation was observed for variants T3 and T4. Both constructs contain large parts of the ligand binding LRR domain of the TSHR. The ability of these chimeras to induce cAMP accumulation after bTSH administration was even lower when compared with rhTSH signaling (Fig. 2A and B, Table 1). A reliable EC_50_ could not be determined due to the fact that the dose response curves did not reach a plateau with a significantly reduced response at the highest bTSH concentration (100 mU/ml) to one third of the wt TSHR signal (Fig. 2B, Table 1). Construct T1 (FSHR-HinR/TSHR) has a higher EC_50_ value compared with the wt TSHR (Fig. 1C, Table 1). Variant F2 with the FSHR-ECD fused to the TSHR TMD did not show significant bTSH mediated intracellular cAMP accumulation (Fig. 1C, Table 1). Interestingly, construct F4 containing 10 LRRs of the FSHR revealed a significant bTSH mediated cAMP production while for construct F3 harboring 11 LRRs of the FSHR only a slight Gs activation after bTSH treatment was observed (Fig. 1C, Table 1).

#### Recombinant human FSH

The most noticeable observation regarding rhFSH signaling is that constructs F2–4 showed a significantly higher cAMP production compared to the wt FSHR (Table 1). Furthermore, construct F3 exhibited a slightly right shifted dose response curve indicated by an EC_50_ of 14.1 ng/ml (wt FSHR: 5.0 ng/ml). Activation of constructs T3 and T4 by rhFSH was not observed (Table 1).

#### Recombinant human FSH analog TR4401

TR4401 showed signaling patterns similar to rhFSH. The use of this ligand resulted in an increase of maximum intracellular cAMP production for constructs F2–4 while no specific activation was observed for constructs T3 and T4. However, the wt TSHR (T0) and the TSHR with the FSHR HinR (T1) showed a pronounced promiscuous stimulation by TR4401 at high concentrations but with no detectable EC_50_ (Table 1).

### ^125^I-bTSH binding

Construct F4, which contains 10 LRRs of the FSHR and the HinR and TMD of the TSHR showed specific ^125^I-bTSH binding of 19% (Table 1). This is in accordance with the observed activation of the Gs pathway after stimulation with bTSH. Impaired ^125^I-bTSH binding was shown for constructs T3 and T4 despite harboring major parts of the LRR domain of the TSHR (Table 1). Constructs F1 and T2 were not characterized for ^125^I-bTSH binding due to the very low cell surface expression.

### ^125^I-hFSH binding

For construct F2 (FSHR-ECD/TSHR) a significant increase in specific ^125^I-hFSH binding to 160% was measured (FSHR set at 100%). In contrast variant F3 showed a decrease of ligand binding to 72% and construct F4 revealed unaltered binding characteristics when compared with the wt FSHR despite a reduced cell surface expression of 45% (Table 1). Constructs F1 and T2-4 were excluded from the ^125^I-hFSH binding experiments because F1 and T2 were not expressed at the cell surface.

### Surface charges at LRR 10 and 11

**Figure 3:**
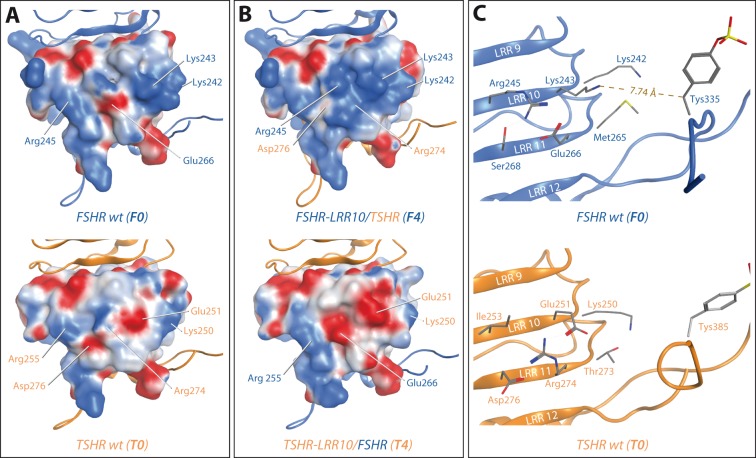
Charge distribution at the C-terminal part of the GPHR LRR domain. A: Marked differences in surface charges exist at the C-terminal part (LRR 10–12) of the LRR domain between the FSHR and the TSHR. While positive charges dominate this area in the FSHR, uncharged and negatively charged patches prevail in the TSHR. B: An accumulation of positive charges can be observed in construct F4 (K242, K243 and R245 from LRR10 of the FSHR and R274 from LRR11 of the TSHR) while the negative charge of D276 from LRR11 of the TSHR is hardly exposed to the protein surface. In the reversed construct T4 prominent negative charges are present at the surface of this area (E251 from LRR10 of the TSHR and E266 from LRR11 of the FSHR). C: side chains of the surface exposed residues located at the beta-sheets of LRR 10 and 11 and position of the sulfated tyrosine in the FSHR (top) and the TSHR (bottom). The distance from the terminal Nitrogen of K243 to the beta carbon of the sulfotyrosine measures 7.74 Å in the crystal structure of the FSHR ectodomain (14).

The analysis of the structural models was focused on the area close to the LRR/HinR transition site due to the lack of structural information on the interplay of ECD and TMD. A comparison of the surface charges of the hormone facing concave side of the LRR domain at LRR 10 and 11 showed that there are marked differences between the FSHR and the TSHR (Figure 3A). While positive charges dominate this area in the FSHR, uncharged and negatively charged patches prevail in the TSHR. The charge distribution of the chimeras with 11 LRRs of the respective receptors (T1, T3 and F1, F3) closely resembles the distribution of the wt receptors with the matching LRR domain. The shorter LRR domain results in an accumulation of negative charges in construct T4 (E251 from LRR10 of the TSHR and E266 from LRR11 of the FSHR) and positive charges in construct F4 (K243 and R245 from LRR10 of the FSHR and R274 from LRR11 of the TSHR) (Figure 3B and C).

## Discussion

The HinR is the least conserved structure within GPHRs and varies in length and amino acid composition [7]. As recently shown by many groups it became obvious that this particular region, which connects the LRRD with the TMD acts as an important ligand binding and signaling component [7], [29]. Despite the recent progress in deciphering large parts of the FSHR HinR structure by Jiang and co-workers [8] and subsequent analysis of TSHR and LHCGR homology models [35], [36], several aspects of receptor structure and function are still unknown. Especially the relative orientation of the ECD (HinR + LRR) to the TMD and in consequence potentially important interactions of both domains are unknown. Based on the relatively low homology for the different HinRs of GPHRs it remains unclear which structural and functional features are conserved between the HinRs of GPHRs and whether there are significant functional receptor specific differences.

### rhFSH and TR4401 do not have significant specific interactions with the HinR

The signaling profile of construct T1 (FSHR-HinR/TSHR) confirmed previously published data showing that bTSH loses its superagonistic activity when the TSHR-HinR is replaced by those of the FSHR or LHCGR [26]–[28]. Interestingly construct F4, which contains the TMD and HinR of the TSHR but major parts of the hormone binding LRR domain of the FSHR displayed significant ^125^I-bTSH binding and activation of the Gs mediated pathway after treatment with bTSH. While the LRR domain is thought to be responsible for ligand specificity [5], binding of bTSH to a receptor with the FSHR LRR domain suggests a major contribution of the HinR to ligand binding. Nagayama and co-workers determined similar bTSH binding properties as shown here for chimera F4, using a chimera of the TSHR with the LHCGR LRR domain (TSH-LHR-9) [28]. This supports the existence of a further binding site for bTSH in the HinR. The stronger interaction of bTSH with the HinR is thought to be mediated by negatively charged residues, which are believed to interact with the four positive lysines in the alpha subunit of bTSH nonexistent in hTSH [12], [13]. Whereas the bTSH binding properties between these two chimeras (TSH-LHR-9 [28] and F4) are comparable major differences occur regarding the signaling capabilities. While bTSH is able to bind to both constructs (TSH-LHR-9 [28] and F4) despite the missing TSHR LRR domain, it only causes Gs activation in chimera F4 (Table 1, Fig. 2C) but not in the TSH-LHR-9 chimera [28]. Strikingly, hCG failed to induce Gs activation at the TSH-LHR-9 construct despite the LHCGR-LRR domain in place [28]. Interestingly, our tested FSH ligands activated chimeras F3 and F4 with almost no differences compared to the wt FSHR. This suggests that only the FSHR-LRR domain is important for specific binding of rhFSH and TR4401. This is supported by a previous report that utilized chimeras between the LHCGR and FSHR. Chimeras with the FSHR LRR domain and the LHCGR TMD comparable to the chimeras F2 and F4, revealed no differences in receptor activation by rhFSH [37]. A study performed with activating antibodies raised against the human FSHR-HinR underlined the importance of this particular structure for activation of the FSHR but showed the limited influence of the FSHR-HinR in hormone binding except for the mandatory sulfated tyrosine [9], [21]. In contrast to rhFSH/TR4401 binding and signaling at the FSHR LRR domain, binding parameters for bTSH are decreased and signaling is severely affected for both ligands at the chimeras with the TSHR LRR domain and the FSHR TMD (T3 and T4, Fig. 1B). This is comparable to the binding and signaling properties of hCG at the TSH-LHR-9 chimera [28].

Based on these data, we conclude that the hormone binding LRR domain of the FSHR contributes nearly exclusively to specific binding of rhFSH and TR4401 due to the fact that changes in Gs activation by these ligands were small or nonexistent in chimeras harboring the FSHR LRR domain. On the other hand, rhTSH, bTSH as shown here and hCG, as shown by Nagayama et al. [28] depend strongly on both structures, the LRR domain and the HinR for correct ligand binding and signal transduction.

### Charge distribution at LRR10 and 11 influences hormone binding

A comparison of the signaling profiles of the chimeras with a different definition for the LRR/HinR transition shows that the selected transition site has a marked impact on the signaling characteristics of the resulting chimeras. Given the high structural similarity of the TSHR and FSHR LRR domain [33], these differences are most likely caused by an altered charge distribution at the transition region rather than global structural changes (Fig. 3). Previous studies and crystallographic data have shown that the charge distribution at the concave face of the LRR domain is responsible for characteristic ligand binding properties [25], [38]. Specific interactions of the ligand's beta chain with the N-terminal LRRs two, three and four as well as the C-terminal LRR nine are considered to be the major determinants of ligand selectivity. But also E251 (K243 in the FSHR) of the TSHR located in LRR10 has been attributed a pivotal role in TSH selectivity and TSHR activation [39], [40] (Fig. 3). In the generated chimeras the introduction of an additional negative charge (E266 from LRR11 of the FSHR in construct T4) (Fig. 1A right panel, Fig. 3A and B) causes a significant impairment of TSH induced signaling. In contrast introduction of an additional positive charge (R274 from LRR11 of the TSHR in construct F4) (Fig. 1A right panel, Fig. 3A and B) results in an improvement of ligand induced signaling. With LRR11 not being in direct contact to the ligand [8], these differences might be caused by allosteric modulation of the LRR binding site or signal transduction towards the TMD. However in the crystal structure this region is also located in close proximity to the sulfation site, which is mandatory for hormone binding [9]–[11] (Figure 3C). The finding that a positive charge has beneficial and a negative charge has detrimental effects on signaling might implicate, that the positive charges at the C-terminal end of the LRR domain interact with the negatively charged sulfotyrosine in the ligand-free state, keeping it in a favorable position for establishing molecular contacts with the ligand upon binding.

### Differences in the HinR-TMD interface among the GPHRs

Recently, it has been shown that the TSHR-HinR can be replaced by the FSHR-HinR and LHCGR-HinR without loss of cell surface expression and signaling [26], [28]. This suggested that the HinR of GPHRs has a common topology and function and might be interchangeable. However, as presented here, introducing the HinR of the TSHR in the background of the FSHR (F1, Fig. 1B) caused a dramatic loss of cell surface expression. This is most likely caused by a loss of important intramolecular interactions or by the introduction of structural clashes between the HinR and the adjacent domains.

The initial chimera (F1) has two transitions between FSHR and TSHR protein sequence, one between LRR11 and the HinR and the other one at the border between the HinR and the TMD. Therefore, the loss of cell surface expression was most likely caused by incompatibility of the TSHR-HinR with the LRR domain, the TMD domain or both adjacent domains of the FSHR.

Replacement of the entire FSHR-ECD by the TSHR-ECD (F2, Fig. 1B) showed a similar phenotype with impaired surface expression. In contrast, keeping the FSHR-HinR/TMD transition intact and only exchanging the FSHR-LRR domain with its TSHR counterpart (constructs F3 and F4, Fig. 1B) resulted in cell surface expression levels even higher than observed for the wt FSHR. This suggests that an incompatibility of the TSHR-HinR with the FSHR-TMD, potentially due to the increased HinR size caused by the C peptide insertion, disturbs protein folding and subsequent cell surface expression. This is contrary to the TSHR-TMD, which tolerates the FSHR-HinR and LHCGR-HinR (T1, Fig. 1B) [26], [28].

So far, it was known that potential interactions between the respective HinRs and the ECLs of TSHR and FSHR are important to restrain the receptors in their inactive state and are further necessary to transmit the extracellular signal to the TMD [18], [19], [22], [41]. These new findings show that besides important signaling properties, there are also significant differences in the HinR-TMD interfaces between the GPHRs.

### Major influences of the TMD on ligand binding and G protein activation

Under the assumption, that the ligand does not directly interact with the TMD [5], results for construct T1 (FSHR-HinR/TSHR), T3 (TSHR-LRR11/FSHR) and T4 (TSHR-LRR10/FSHR) (Fig. 1B) suggest that the FSHR HinR in conjunction with the TSHR TMD in construct T1 behaves differently than in conjunction with the FSHR TMD in constructs T3 and T4. In the construct with the FSHR HinR and the TSHR TMD (T1) rhTSH shows unaltered signaling and bTSH reduced potency when compared with the wt TSHR [26]. In contrast the constructs with the FSHR HinR and the FSHR TMD (T3 and T4) showed a drastic reduction in efficacy and potency of the rhTSH and bTSH mediated cAMP production. These findings lead us to hypothesize that the TMDs, especially the ECLs, of GPHRs are major contributors for the conformation of the respective HinRs. In the case of construct T1 the TSHR TMD induces a conformation of the FSHR HinR keeping rhTSH signaling and very likely also binding unaffected. However, some structural features of the TSHR HinR involved in bTSH binding are apparently missing in the FSHR HinR indicated by significant changes in bTSH mediated signaling. The strong impairment in TSH signaling due to decreased ligand binding, despite the LRR domain of the TSHR, observed for constructs T3 and T4, is most likely due to the FSHR ECLs inducing a conformation of the FSHR HinR similar to the wt FSHR, which is most likely unfavorable for rhTSH/bTSH binding.

Radical effects on ligand binding specificity and signaling due to changes in the TMD were shown for FSHR mutations, which have been identified as the cause of ovarian hyper stimulation syndrome (OHSS) [42], [43]. The described FSHR variants were found in the TMD and showed constitutive activity for the Gs pathway as well as promiscuity towards rhTSH and hCG. The authors of these studies suggested that the mutations change the receptor conformation on the level of the transmembrane helices, which would lead (I) to the ligand independent activation of intracellular G proteins and (II) to the release of inhibitory constraints between the HinR and the TMD allowing the FSHR to bind different hormones like hCG and also rhTSH [42], [43]. Furthermore, these interpretations and our results imply that the conformation of the transmembrane helices and their adjacent ECLs can have a strong impact on events at the extracellular site, which most likely also involves conformational changes of the HinR and consequently changes in ligand specificity. Next to these effects the TMD apparently also dictates G protein coupling. This is suggested by only about half the maximal stimulation of constructs including the FSHR TMD as compared with constructs bearing the TSHR TMD.

## Conclusions

In summary, our results show that (I) the adjacent domains (LRR domain, TMD) dictate functional characteristics of the HinR like binding and signaling. (II) The HinRs of GPHRs have specific structural requirements, indicated by missing specific interactions or additional structural clashes at the HinR/TMD interface *e.g.* between the TSHR HinR and FSHR TMD resulting in a loss of cell surface expression. (III) bTSH is more sensitive to changes in the HinR and has stronger interactions with the C-terminal part of this particular ECD structure, when compared with rhTSH. IV) Differences in charge distributions in the C-terminal region of the LRR domain affect binding and signaling by TSH or FSH. Introduction of an additional positive charge improved signaling for FSH, whereas an additional negative charge had the opposite effect for TSH activation.

The HinRs of GPHRs share some structural and functional characteristics *e.g.* the mandatory sulfotyrosine [9], [10] or the signaling relevant serine at the end of LRR11 (TSHR: 281, FSHR: S273, LHCGR: S277) [44], [45]. However, we provide new evidence that the HinRs of TSHR and FSHR have their own ligand specificities, which depend mostly on the correct interplay with the adjacent TMD.
